# Design and validation of a food frequency questionnaire to assess the dietary intake for adults in pastoral settings in Northern Tanzania

**DOI:** 10.1186/s13104-021-05692-8

**Published:** 2021-07-17

**Authors:** Ahmed Gharib Khamis, Akwilina Wendelin Mwanri, Julius Edward Ntwenya, Mbazi Senkoro, Katharina Kreppel, Bassirou Bonfoh, Sayoki Godfrey Mfinanga, Gideon Kwesigabo

**Affiliations:** 1 Department of Epidemiology and Biostatistics, Muhimbili University of Health and Allied Sciences, Dar-Es-Salaam, Tanzania; 2 Department of Food Technology, Nutrition and Consumer Sciences, Sokoine University of Agriculture, Morogoro, Tanzania; 3 Department of Public Health, The University of Dodoma, Dodoma, Tanzania; 4 National Institute for Medical Research, Muhimbili Medical Research Centre, Dar-es-Salaam, Tanzania; 5 School of Life Sciences and Bio-Engineering, Nelson Mandela African Institution of Science and Technology, Arusha, Tanzania; 6 Department of Environmental Health and Ecological Sciences, Ifakara Health Institute, Dar-es-salaam, Tanzania; 7 Centre Suisse de Recherches Scientifiques en Côte D’Ivoire, Abidjan, Côte d’Ivoire

**Keywords:** Relative validity, Food frequency questionnaire, Diet recall, Pastoralists

## Abstract

**Objective:**

Food frequency questionnaires are widely used as a dietary assessment tool in nutritional epidemiology to determine the relationship between diet and diseases. In Tanzania, there are several cultural variations in food intake which makes it necessary to design and validate a culture-specific food frequency questionnaire (CFFQ). Therefore, we designed a 27-items CFFQ and examine its validity in pastoral communities. Validity of CFFQ was assessed by comparing nutrient intake estimated from the CFFQ against the average from two 24-h diet recall (2R24). Spearman’s correlation coefficients, cross classification and Bland–Altman’s methods were used to assess the validity of CFFQ.

**Results:**

A total of 130 adults aged 18 years and above completed both CFFQ and 2R24. Correlation coefficients between CFFQ and 2R24 ranged from low (r = − 0.07) to moderate (r = 0.37). The correlation coefficients were moderately significant for kilocalories (r = 0.31, *p* < 0.001), carbohydrate (r = 0.33, *p* < 0.001), magnesium (r = 0.37, *p* < 0.001), and iron (r = 0.34, *p* < 0.001). On average, about 69% of participants were correctly classified into the same or adjacent quartile of energy and nutrient intake, while 9% were misclassified by the CFFQ. Bland–Altman’s plot demonstrated that the CFFQ had acceptable agreement with the 2R24.

**Supplementary Information:**

The online version contains supplementary material available at 10.1186/s13104-021-05692-8.

## Introduction

The World Health Organization (WHO) estimated that approximately 1.7 million deaths worldwide are attributable to an unhealthy diet [1]. Unhealthy diet is a cornerstone of several non-communicable diseases (NCDs) such as hypertension, type 2 diabetes and cancer [2–4]. Tanzania has made significant achievement in the reduction of diet-related NCDs despite the levels being high [5, 6]. Evaluating for dietary intake of the population is important because it help to determine the nutritional status and understand the association with diet-related diseases such as NCDs.

Literature suggests that the use of food frequency questionnaire (FFQ) is more appropriate for measuring habitual intake of foods and nutrients than alternative methods such as 24-h diet recall and food records [7, 8]. These questionnaires are relatively cheap to administer in population-based studies [7, 9, 10]. Because a FFQ does not necessarily estimate the actual amount of food intake, their validity needs to be evaluated. There is no ‘gold standard’ for validation of FFQ, but commonly the estimated intake from FFQ is compared against the intake from other dietary assessment methods [11].

To date, numerous studies have been devoted to assess the validity of FFQs before their application in Tanzania and elsewhere [12–15]. However, the applicability of these questionnaires for use in pastoral settings is limited. Pastoral livelihoods depend on animal production, and they have different dietary habits from that of urban populations. It is therefore important to design a FFQ that contains appropriate food items suitable for use in pastoral communities in order to correspond to the prevailing food culture [11]. In this study, we explain the design of a culture-specific food frequency questionnaire (CFFQ) as part of a study that investigating the influence of dietary factors on NCDs in pastoral communities in Tanzania. This study aims to assess the relative validity of a CFFQ by comparing the dietary intake measured from CFFQ against the average of two 24-h diet recalls (2R24).

## Main text

### Materials and methods

#### Study participants

This was a cross-sectional study involving adults aged 18 years or older who are permanent residents of Monduli district of Arusha Region (northern Tanzania). The majority of the population in the district identifies as traditional Maasai pastoralists (97%) and few agro-pastoralists who predominantly live in rural areas. We conducted the face to face interviews in a “boma” setting. A “boma” is a collection of households enclosed by a tree branches to protect animals from intruders and predators. A total of 15 “bomas” were selected from previously sampled villages [16]. Households were randomly selected from each “boma” using a random start approach until the required number was reached. The number of participants was set at 150 after considering the recommended sample size of 100 for the validation study by Cade et al. [7], and after taking into account the loss to follow-up.

#### Development of the CFFQ

A culture-specific food frequency questionnaire (CFFQ) was designed from the food consumption data of one day 24-h dietary recall representative for the study population as described previously in detail [16]. By using stepwise multiple regression analysis, food items in the model that explained 90% (R^2^ ≥ 90) of the between-person variability of total energy, carbohydrates, protein and fats were considered in the final CFFQ. About 42 foods and beverages were included in the initially developed CFFQ. We pilot tested these food items in a sub-sample (n = 31) in order to identify foods that were consumed rarely and on special occasions. None of these subjects were included in the final validation study. Finally, this resulted in a CFFQ of 27 food items (Additional file 1). In the CFFQ, all participants were required to indicate how many times they consumed each food item in the past month. The frequency of consumption was measured by selecting one of the following nine options: (1) never; (2) 1–3 per month (3) once per week; (4) 2–4 times per week; (5) 5–6 times per week; (6) once per day; (7) 2–3 times per day (8) 4–5 times per day (9) 6 + times per day [15]. Portion sizes for food items and mixed dishes were defined based on the most commonly consumed portion sizes and mixed dishes. Pictures of utensils and photographs were also used during the interview to assist the participants.

#### Validation of the CFFQ

All participants were requested to complete two 24-h diet recalls for non-consecutive days (2R24) as reference method for validation [11]. We administered the first 24-h diet recall with the CFFQ during the first visit. A second 24-h diet recall was repeated on the same participant after three to four weeks. All participants were asked to recall foods and beverages consumed on the previous day. This included quantitative response alternatives of the portion size consumed (bowl, plate, saucer, units, glasses or cups). As foods are often eaten as composite dishes, we asked them to estimate amounts of individual components eaten.

#### Estimation of energy and nutrient intake

Nutrient values were taken from the Tanzania Food Composition Table (TFCT) [17]. The TFCT provides the amounts of each nutrient per 100 g for individual foods, beverages and for mixed dishes. For other local foods items that are absent in TFCT, similar foods were selected based on the nutritional composition of that food. For the CFFQ, the frequency responses were converted into number of servings per day (for-example, once per week = 0.143 servings per day) and multiplied by portion size [18]. For every 100 g of food, we estimated the daily intake of energy and nutrients.

#### Data analysis

Median and interquartile ranges (IQR) for dietary energy and nutrient intake were compared using a Wilcoxon signed rank test [19]. Macronutrients were separately adjusted for total energy intake using the nutrient density method as a percentage of energy. We used the Spearman’s correlation coefficients with 95% confidence intervals (CI), cross-classification and Bland–Altman’s methods to assess the validity of CFFQ. Weighted Kappa (k) values were calculated and interpreted as follows: > 0.80 indicates very good agreement, 0.61–0.80 good agreement, 0.41–0.60 moderate agreement, 0.21–0.40 fair agreement, and < 0.20 poor agreement [20]. The Bland–Altman’s method was used to visualize the agreement between CFFQ and 2R24. The differences in nutrient intake from the two dietary methods (CFFQ −2R24) were plotted against the mean of nutrient intake from the two methods ((CFFQ + 2R24)/2) [7]. Analysis was performed using Stata 16 (Texas, USA) and SPSS version 23 (Armonk, New York, USA).

### Results

A total of 130 participants completed both CFFQ and 2R24. Twenty participants (n = 20) were lost to follow-up. Participants had a mean (± SD) age of 35.3 (± 17.0) years, and 98 (75.3%) were females. Table 1 shows that there was a significant difference in median daily intake estimated by CFFQ and 2R24 (*p* < 0.05). Moreover, low (*r* = -0.07) to moderate (*r* = 0.37) Spearman’s rank correlation coefficients were found between the CFFQ and average of 2R24. Correlation coefficients were moderately significant for kilocalories (r = 0.31, *p* < 0.001) carbohydrate (r = 0.33, *p* < 0.001), magnesium (r = 0.37, *p* < 0.001), and iron (r = 0.34, *p* < 0.001).

**Table 1:** Comparison of dietary energy and nutrients intake estimated from two 24-h diet recalls and the culture-specific food frequency questionnaires among pastoralists (N = 130)

Energy and nutrients	2R24		CFFQ		Spearman’s rank correlation	
	Median	IQR	Median	IQR	*r*	95% CI
Energy (kcal/d)	2005.5	2289.2–1446.9	2403.9	4682.3–1544.4	0.31***	0.15; 0.46
Macronutrients						
Carbohydrates (g/d)	331.2	390–253.6	370.4	686.4–252.1	0.33***	0.18; 0.48
Carbohydrates (% of energy)	68.1	78.4–60.3	62.2	66.7–58.1	− 0.09	− 0.27; 0.07
Fat (g/d)	43.3	74.4–20.7	63.7	147.2–41.5	0.19*	0.02; 0.35
Fat (% of energy)	21.7	30.2–12.2	27.5	30.6–22.4	− 0.04	− 0.17; 0.13
Protein (g/d)	44.1	54–34.5	67.9	150.1–45.3	0.16	− 0.02; 0.33
Protein (% of energy)	9.4	10.9–8.5	12.6	13.8–11.4	− 0.24	− 0.41; 0.05
Cholesterol (g/d)	14.1	37.6–0	61.5	125.4–37.1	− 0.12	− 0.3; 0.06
Fiber (g/d)	29.5	39.8–20.9	40.5	99.8–29.7	0.26**	0.10; 0.44
Micronutrients						
Calcium (mg/d)	170.2	349.0–80.3	573.4	1416.6–290.8	− 0.07	− 0.25; 0.14
Phosphorous (mg/d)	1052	1322.7–818.3	1703.4	3593.9–1097.4	0.28**	0.11; 0.44
Magnesium (mg/d)	444.5	606.3–310.8	619.7	1122.5–394.4	0.37***	0.22; 0.51
Potassium (mg/d)	1964.6	2615–1379.1	3443.6	6769.8–2315.7	0.22**	0.07; 0.39
Sodium (mg/d)	417.4	756–166.8	625.9	1271.4–417.3	0.12	− 0.05; 0.29
Iron (mg/d)	14.1	19.2–8.8	18.1	43.1–13.0	0.34***	0.18; 0.50
Zinc (mg/d)	8.2	10.3–6.1	9.5	20.5–6.5	0.21*	− 0.01; 0.35
Vitamin A (µg RE/d)	98.7	184.5–45.6	694.9	911.1–269.9	− 0.12	− 0.28; 0.05
Vitamin E (µg/d)	2.4	3.4–1.4	7.7	11.7–4.6	0.16*	0.02; 0.35
Vitamin C (mg/d)	10.1	43.6–5	75.6	100.0–49.6	− 0.06	− 0.23; 0.13
Vitamin B2 (mg/d)	1.2	1.4–0.8	1.9	3.7–1.0	0.23*	0.07; 0.40
Vitamin B6 (mg/d)	1.3	1.5–1	1.9	3.1–1.2	0.22*	0.03; 0.39
Vitamin B12 (µg/d)	0.4	1.3–0	1.5	3.4–0.8	− 0.14	− 0.31; − 0.04

IQR = Interquartile range; Spearman correlations were used to assess the relationship of energy and nutrient intakes; Correlation was significant at *P ≤ 0.05, **P < 0.01, **P < 0.01 (2-tailed)

The percentage of participants correctly classified into the same quartile ranged from 20% (sodium) to 37.7% (kilocalories). On average, about 69% of participants were correctly classified within one quartile which ranges from 53.8% (vitamin A) to 79.2% (iron and magnessium). The proportion of participants classified into opposite quartile was on average 9.6%. Most of the nutrients have shown a significant fair classification agreement with acceptable kappa statistic (k) in magnesium (k = 0.4, p < 0.001), kilocalories (k = 0.34, p < 0.001), carbohydrate (k = 0.34, p < 0.001), iron (k = 0.38, p < 0.001) and phosphorous (k = 0.3, p < 0.001) as shown in Table 2.

**Table 2:** Agreement analysis by cross-classification into quartiles of dietary energy and nutrients derived from two 24-h diet recall and the culture-specific food frequency questionnaire among pastoralist (N = 130)

Energy and nutrients	Classified into same quartile (%)	Classified into same or adjacent quartile (%)	Classified into opposite quartile (%)	Weighted Kappa (k)
Energy (kcal/d)	37.7	75.4	5.4	0.34***
Macronutrients				
Carbohydrates (g/d)	34.6	76.1	5.3	0.34***
Fat (g/d)	29.2	73.1	9.2	0.20*
Protein (g/d)	31.5	70.0	9.2	0.18*
Cholesterol (g/d)	20.7	57.7	14.6	− 0.09
Fiber (g/d)	33.8	75.4	7.7	0.28**
Micronutrients				
Calcium (mg/d)	20.7	62.3	13.8	− 0.03
Phosphorous (mg/d)	33.1	75.4	6.9	0.30***
Magnesium (mg/d)	31.5	79.2	3.8	0.40***
Potassium (mg/d)	30.7	73.1	10.0	0.20*
Sodium (mg/d)	20.0	66.1	10.7	0.04
Iron (mg/d)	37.6	79.2	5.3	0.38***
Zinc (mg/d)	30.7	71.5	6.1	0.25**
Vitamin A (µg RE/d)	23.8	53.8	13.1	− 0.1
Vitamin E (µg/d)	30.0	68.4	13.1	0.1
Vitamin C (mg/d)	21.5	57.7	16.1	− 0.07
Vitamin B2 (mg/d)	31.5	70.0	6.1	0.24**
Vitamin B6 (mg/d)	26.9	72.3	12.3	0.16
Vitamin B12 (µg/d)	23.8	58.4	13.8	− 0.09
Average	28.9	69.2	9.6	

Kappa statistics (k) is significant at *P ≤ 0.05, **P < 0.01, **P < 0.01 (2-tailed)

**Fig. 1:**
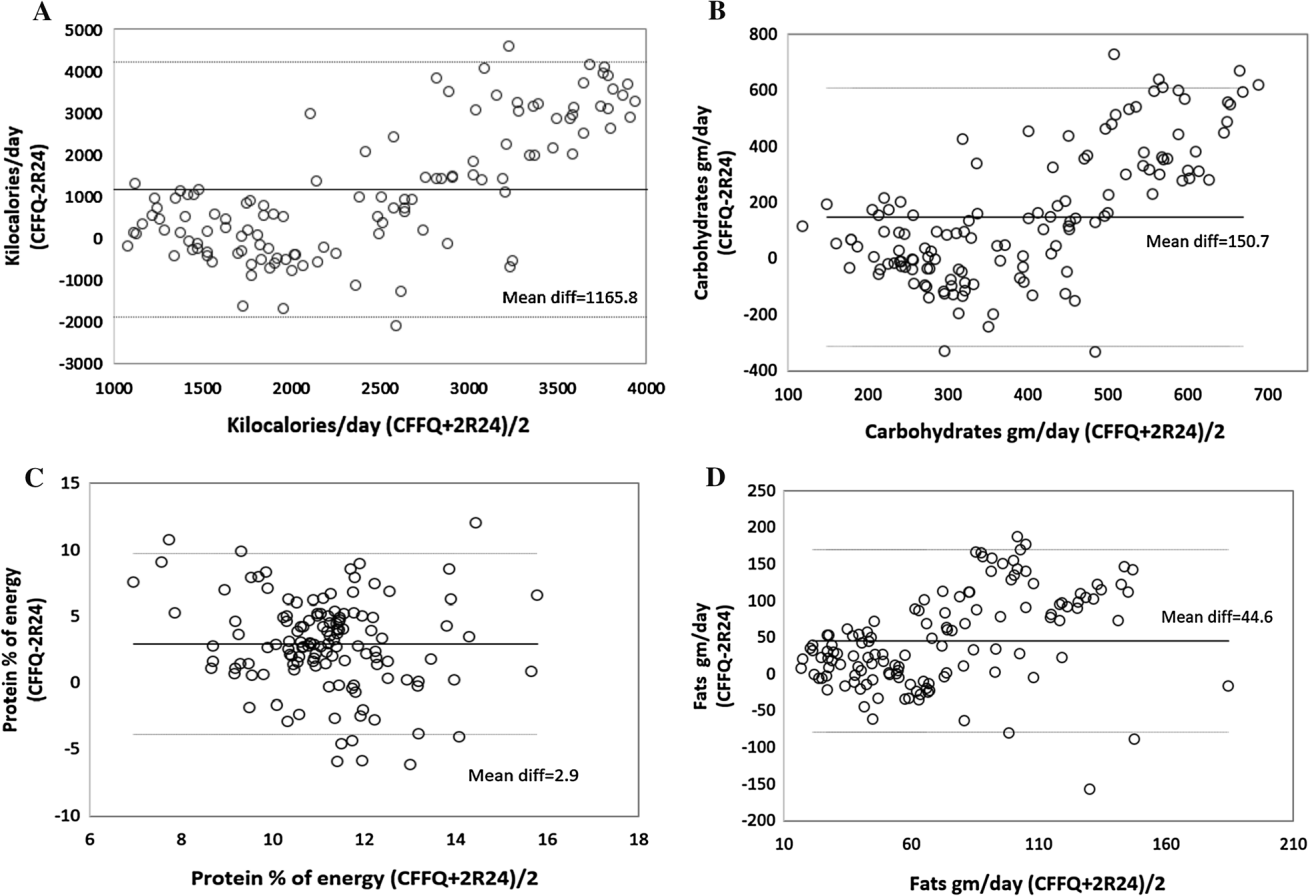
Bland–Altman’s plots showing the agreements between CFFQ and 2R24 for (**A**) Kilocalories (**B**) Carbohydrates (**C**) Protein (% of energy) and (**D**) Fats

Moreover, Bland–Altman’s analysis shows that the CFFQ systematically overestimated kilocalories, carbohydrates, % protein and fats. There is, however, an acceptable level of agreement between both methods as most points were close to the mean and distributed within the 95% limit of agreement (LOAs), while very few (less than 10%) were outside the LOAs (Fig. 1).

### Discussion

In this study, we examined the relative validity of a CFFQ in pastoral communities in northern Tanzania. We found that the correlation coefficients between CFFQ and 2R24 range from low to moderate. Importantly, there was acceptable ranking of CFFQ in relation to 2R24. Therefore, our CFFQ has shown a low to moderate relative validity in measuring absolute nutrient intake, and found to be acceptable in classifying individuals according to dietary consumption in pastoral communities.

While the correlation coefficients reported here were small, this is consistent with the previous FFQ validation studies conducted in other parts of Tanzania [12, 18]. Moreover, our CFFQ tends to overestimate intake of energy and nutrients in relation to 2R24. Overestimation from CFFQ was expected because participants were asked to report the frequency of consumption of a finite list of food items over one month, while in 24-h diet recall participants reported the actual food intake for previous day [20]. In comparison, overestimation from FFQ has been widely reported in many validation studies [21–23].

As previously suggested, when validating a FFQ—correlation coefficients should be at least 0.3 [7, 24]. This study indicating that the designed CFFQ may be capable of estimating absolute intake of kilocalories, carbohydrate, magnesium and iron. This may be due to the fact that these nutrients are available in most types of foods that form part of the daily meal in this community. We also found significant, however a small correlation for some micronutrients. The small correlation can be explained by little consumption of micronutrients rich foods such as fruits and vegetables in the study population. The small correlation of micronutrients in this study are somehow comparable with those reported in other validation studies in Tanzania [18], Botswana [25] and Bangladesh [26].

We found that agreement in ranking of participants was significant for most nutrients. This important finding demonstrates that the CFFQ is satisfactory in ranking of individuals, and this was the main purpose of developing this FFQ. The percentage of disagreement was on average 9.6%, which is acceptable level as shown by Masson et al. [24]. Similar findings have been reported from validation studies elsewhere [27, 28]. Nevertheless, our CFFQ is relatively shorter than other previous developed FFQs in African countries such as South Africa [29] and Mali [30], but longer compared to that for adults in rural areas of Rwanda [31]. To our knowledge, this is the first report on validation of a CFFQ in Tanzania.

This study has some strength. By collecting a repeated 24-h diet recalls it may ensure the accuracy of the dietary data. We are confident that the food list in the designed CFFQ covers more than 90% of the typical daily diet consumed in this community, since the development of the food list was based on pilot testing of CFFQ and that the diet of population is relatively homogeneous and simple.

In conclusion, the designed CFFQ can be useful in ranking participants based on their food consumption in pastoral communities. The CFFQ has a low to moderate relative validity for measurements of absolute nutrients intake due to overestimation. This however provides encouragement to the conducting of nutritional epidemiological studies using CFFQ in rural and hard to rich population like “Maasai” pastoralists.

## Limitations

There are some limitations in this study. Our CFFQ was not very exhaustive and consists of food items that are regularly consumed in the community. However, the main purpose was to develop a short and CFFQ that can be used to assess the dietary habits of adults in the community. In addition, CFFQ and 2R24 are both dependent on memory [14], therefore, we cannot rule out the possibility of over and under-estimation from participants. As opposed to conventional recommendations for FFQ validation studies [7], it was not possible to validate against 7-days food records as this may require high literacy.

## Supplementary Information

**Additional file 1:** Culture-specific food frequency questionnaire to assess the dietary intake of the pastoralist in Monduli district, Northern Tanzania.

## Data Availability

The data used to support the findings of this study are available from authors upon special request.

## Abbreviations

2R24: Two days 24-h diet recalls
CFFQ: Culture specific food frequency questionnaire
FFQ: Food frequency questionnaire
NCDs: Non-communicable diseases
