# Supplementary material for: Design and validation of a food frequency questionnaire to assess the dietary intake for adults in pastoral settings in Northern Tanzania
Source: BMC Res Notes. 2021 Jul 17;14:274. doi: 10.1186/s13104-021-05692-8 (PMC8285883; doi:10.1186/s13104-021-05692-8)
Supplement: Supplementary file 1 — Additional file 1: Culture-specific food frequency questionnaire to assess the dietary intake of the pastoralist in Monduli district, Northern Tanzania. [file 13104_2021_5692_MOESM1_ESM.docx]

**Culture-specific food frequency questionnaire to assess the dietary intake of the pastoralist in Monduli district, Northern Tanzania**

**Question:** How often you eat at least ONE portion of the following foods and drinks in the past 30 days?

**NOTE:** Please pick only one frequency for each food.

About serving sizes: Example 1 serving spoon = ½ cup. If you consume 2 servings of a food once a week, then you will check “2-4 per week.”

| Code | Name of food | Serving size | Never | 1-3 per month | once per week | 2-4 times per week | 5-6 times per week | once per day | 2-3 times per day | 4-5 times per day | 6+ times per day |
| --- | --- | --- | --- | --- | --- | --- | --- | --- | --- | --- | --- |
| F01 | Mixed porridge (*Uji*) | 1 cup |  |  |  |  |  |  |  |  |  |
| F02 | Stiff porridge (*Ugali*) | 1 plate |  |  |  |  |  |  |  |  |  |
| F03 | Maize and beans, mixed dish (*Makande*) | 1 plate |  |  |  |  |  |  |  |  |  |
| F04 | Beans, alone | 1/2 plate |  |  |  |  |  |  |  |  |  |
| F05 | Milk | 1 cup |  |  |  |  |  |  |  |  |  |
| F06 | Yoghurt | 1 cup |  |  |  |  |  |  |  |  |  |
| F07 | Milk and maize, mixed dish (*Loshoro*) | 1 cup |  |  |  |  |  |  |  |  |  |
| F08 | Tea with sugar | 1 cup |  |  |  |  |  |  |  |  |  |
| F09 | Beef | 1 palm-size |  |  |  |  |  |  |  |  |  |
| F10 | Goat | 1 palm-size |  |  |  |  |  |  |  |  |  |
| F11 | Meat soup, mixed dish | 1 bowl |  |  |  |  |  |  |  |  |  |
| F12 | Bread (*Chapatti*) | 1 piece |  |  |  |  |  |  |  |  |  |
| F13 | Fruit juice, mixed | 1 250 ml glass |  |  |  |  |  |  |  |  |  |
| F14 | Mango | 1 medium piece |  |  |  |  |  |  |  |  |  |
| F15 | Spinach | 1/2 plate |  |  |  |  |  |  |  |  |  |
| F16 | Fried donut | 1 piece |  |  |  |  |  |  |  |  |  |
| F17 | Banana cooked with meat (*Mtori*) | 1 bowl |  |  |  |  |  |  |  |  |  |
| F18 | Cooked cassava | 1 plate |  |  |  |  |  |  |  |  |  |
| F19 | Sweet potato | 1 plate |  |  |  |  |  |  |  |  |  |
| F20 | Banana ripe | 1 medium piece |  |  |  |  |  |  |  |  |  |
| F21 | Vegetables, mixed dish | 1/2 plate |  |  |  |  |  |  |  |  |  |
| F22 | Cabbage | 1 plate |  |  |  |  |  |  |  |  |  |
| F23 | Local brew | 1 bowl |  |  |  |  |  |  |  |  |  |
| F24 | Sugary beverages (e.g *Coke, Fanta*) | 1 350 ml bottle |  |  |  |  |  |  |  |  |  |
| F25 | Milk tea with sugar | 1 cup |  |  |  |  |  |  |  |  |  |
| F26 | Green peas, alone | 1/2 plate |  |  |  |  |  |  |  |  |  |
| F27 | Other wild green vegetables | 1/2 cup |  |  |  |  |  |  |  |  |  |
